# Benign Presentation Following Massive Deutetrabenazine Overdose

**DOI:** 10.7759/cureus.12886

**Published:** 2021-01-24

**Authors:** Oluseyi Obadeyi, James H Paxton, Sarkis Kouyoumjian

**Affiliations:** 1 Emergency Medicine, Wayne State University School of Medicine, Detroit, USA; 2 Emergency Medicine, Wayne State University Detroit Medical Center, Detroit, USA

**Keywords:** deutetrabenazine, overdose, toxicology, side effects, toxicity, tardive dyskinesia

## Abstract

Deutetrabenazine (DTBZ) (Austedo®) is a novel vesicular monoamine transporter 2 (VMAT2) inhibitor, which acts by blocking dopamine release and other monoamines from neuronal vesicles. Although this drug is considered the first-line treatment for tardive dyskinesia (TD), VMAT2 inhibition has also been shown to improve patients’ symptoms with Huntington’s disease-induced chorea. We present the case of a 59-year-old woman with a history of TD, who presented to the emergency department following massive DTBZ ingestion. The relative paucity of other overdose symptoms further supports the manufacturer’s claims of a low side effect profile for this drug in overdose. Although DTBZ demonstrates an excellent safety profile, emergency physicians should be aware of the potential side effect of DTBZ overdose, in addition to other known side effects of this novel drug.

## Introduction

Deutetrabenazine (DTBZ) (Austedo®) is a novel vesicular monoamine transporter 2 (VMAT2) inhibitor that blocks dopamine release and other monoamines from neuronal vesicles. Although this drug is considered the first-line treatment for tardive dyskinesia (TD), VMAT2 inhibition has been shown to improve patients’ symptoms with Huntington’s disease (HD)-induced chorea [1]. TDs are involuntary movements of the tongue, lips, face, trunk, or extremities associated with and often attributed to long-term use of dopaminergic antagonist medications [1]. The unique movements of TD are repetitive, purposeless, and involuntary and can often cause functional impairment. We present the case of a 59-year-old woman with a history of TD, who presented to the Emergency Department (ED) following massive DTBZ ingestion. The patient had an benign course.

## Case presentation

A 59-year-old female with a history of hypertension, type 2 diabetes, hypothyroidism, TD, Parkinson’s disease, schizophrenia, bipolar disorder, and ischemic stroke presented to the ED with her daughter for DTBZ overdose. Her daughter stated that the patient ingested 20 tablets of 12 mg DTBZ (Table 1) approximately more than eight hours before presenting to the ED. She denied wanting to hurt herself and having any history of a suicide attempt. Our patient stated that she took the medication because she thought it was a pain medication that would relieve a left lower extremity pain that she sustained from hitting her left lower leg on a bed frame. She admitted to feeling depressed, and her only complaint was that she felt drowsy. She had no other symptoms or complaints. The patient denied headache, tinnitus, nausea, vomiting, chest pain, shortness of breath, or any other symptoms.

Her vital signs on arrival revealed a temperature of 98.8°F, blood pressure of 162/104 mmHg, heart rate of 74 beats per minute, respiration of 20 breaths per minute, and oxygen saturation (SpO_2_) of 96% on room air. Physical examination revealed an alert and oriented female who was in no acute distress. She had clear bilateral lung sounds, non-labored respirations with a regular heart rate, and rhythm. Aside from tremors noted on her right hand, she had unremarkable sensory, strength, and deep tendon reflexes in all extremities.

**Table 1:** Patient’s outpatient medications prior to overdose presentation Mg: milligrams, mCg: microgram, Cap: capsule

Drug	Dose
Aspirin	81 mg by mouth, daily
Atorvastatin	40 mg by mouth, daily
Carvedilol	12.5 mg by mouth, twice daily
Clopidogrel	75 mg by mouth, daily
Deutetrabenazine	12 mg by mouth, daily
Docusate	One Cap 100 mg by mouth, twice daily
Hydralazine	75 mg by mouth, three times daily
Levothyroxine	125 mCg by mouth, daily
Nifedipine	Tab 90 mg by mouth, daily
Quetiapine	Tab 50 mg by mouth, before bed

Electrocardiogram demonstrated normal sinus rhythm. She had a narrowed anion gap, and her complete blood count and electrolyte levels were within normal limits. Her urine drug screen for amphetamines, barbiturates, benzodiazepines, cannabinoids, cocaine metabolite, methadone, and opiates was negative.

The toxicology service was consulted and recommended 23 hours of telemetry observation to monitor for QT prolongation. The patient had an uneventful overnight course. The next morning, her orofacial TD was present, and neurology was consulted. Neurology recommended psychiatry consult to evaluate for suicidal ideation and outpatient follow-up to discuss the resumption of DTBZ. From a medical standpoint, the patient had an unremarkable hospital course. She was medically cleared by the neurology and inpatient team on day two of her admission. She was admitted for three days, and her length of stay was prolonged because she reported to the psychiatry team that she would like to be placed in a group home facility instead of living with her daughter at home. The patient was finally discharged home with her daughter after changing her mind about desiring a group home placement.

## Discussion

Before the introduction of VMAT2 inhibitors, TD was believed to be an irreversible side effect of dopamine antagonism. Neuroleptics and antiemetic dopamine blocking agents cause TD as a side effect of their clinical use. First- and second-generation antipsychotic treatments have been linked with a 5.5% and 3.9% yearly incidence rate of TD development, respectively [1]. Although the pathophysiology of TD development is poorly understood, it is thought to involve the upregulation and sensitization of the dopamine D2 receptor as a result of the prolonged blockade [2].

DTBZ functions as a reversible VMAT2 inhibitor. The US Drug Administration (FDA) approved it in August 2017 for the treatment of TD in adults. The medication is also approved to treat HD-induced chorea and Tourette syndrome [3-5]. Tetrabenazine (TBZ) is the archetype VMAT2 inhibitor and was used for decades to manage hyperkinetic movement disorders before developing other VMAT2 inhibitors like DTBZ and valbenazine (Ingrezza®). The substandard pharmacokinetic profile and lack of FDA approval other than its use for HD chorea limited the use of TBZ in the US. Valbenazine and DTBZ addressed some of the deleterious pharmacokinetic profile of TBZ. Although TBZ and DTBZ are structurally similar, the substitution of deuterium for hydrogen in strategic locations of TBZ resulted in a compound with better pharmacokinetics and safety profile [6-8]. At approximately half the dose of TBZ, DTBZ offers a comparable total exposure time with a longer half-life and at a lower maximum serum concentration [8].

Although it is safer than TBZ, DTBZ still has the potential to cause serious side effects. A 12-week trial examining patients with HD revealed that somnolence was the most common dose-limiting side effect of DTBZ [9]. The other adverse effects noted in TD patients include nasopharyngitis, insomnia, depression, and akathisia [3,9]. DTBZ can induce QT prolongation in patients who are CYP2D6 poor metabolizers or consuming a potent CYP2D6 inhibitor. DTBZ also has the potential to cause hyperprolactinemia and neuroleptic malignant syndrome (NMS) [3]. Patients with NMS present with fever, muscle rigidity, and altered mental status with autonomic changes. The novelty of DTBZ demonstrates the lack of sufficient information on the potential signs and symptoms of DTBZ overdose.

In this case, the patient had no significant adverse effect from a substantial DTBZ consumption at the time of initial presentation. DTBZ for the management of TD appears to be well-tolerated. The recommended starting dose of DTBZ for the management of TD is 6 mg/day, and it can be titrated up to a recommended maximum dose of 48 mg per day. Previous studies started DTBZ at 12 mg/day (6 mg twice daily) with weekly titration of 6 mg/day for up to six weeks until the maximum 48 mg per day or adequate TD control was reached, barring the occurrence of a severe side effect [7]. Due to the ability of TBZ to cause QT interval prolongation, it is recommended that patients at risk of developing QT prolongation have their QT interval assessed before and after increasing their total dose above 24 mg per day [3].

The diagnosis of DTBZ overdose can only be made through history taking and clinical presentation. Management of DTBZ overdose includes discontinuation of DTBZ, symptomatic treatment, and medical monitoring. DTBZ metabolites are primarily metabolized by CYP2D6 and virtually eliminated renally [3]. Although she had ingested ten times her prescribed daily dose of DTBZ, the patient described in this case report had a mild symptom. The only symptom that she complained of was somnolence, which is the most common adverse effect of DTBZ in patients [3,7,9]. The reported half-life of the active metabolites of DTBZ is nine to ten hours [3]. Considering the substantial amount of DTBZ the patient ingested, a noteworthy observation was that the patient’s orofacial TD manifested the following morning after her admission 24 hours after DTBZ ingestion.

## Conclusions

DTBZ overall has an excellent safety profile with tolerable side effects when utilized for the management of TD. Because of its novelty, limited studies have investigated the potential clinical presentation of DTBZ poisoning. Our patient had a mild symptom following massive DTBZ ingestion. Although DTBZ demonstrates an excellent safety profile, emergency physicians should be conscious of its potential to cause fetal abnormalities like QT prolongation and NMS. Consultation with a medical toxicologist and heart monitoring for QT prolongation is recommended.
